# Analysis of Questionnaire for Traditional Medicine and Development of Decision Support System

**DOI:** 10.1155/2014/974139

**Published:** 2014-01-29

**Authors:** Kotoe Katayama, Rui Yamaguchi, Seiya Imoto, Kenji Watanabe, Satoru Miyano

**Affiliations:** ^1^Human Genome Center, Institute of Medical Science, University of Tokyo, 4-6-1 Shirokanedai, Minato-ku, Tokyo 108-8639, Japan; ^2^Center for Kampo Medicine, Keio University School of Medicine, 35 Shinano-machi, Shinjuku-ku, Tokyo 160-8582, Japan; ^3^Faculty of Environment and Information Study, Keio University, 5322 Endo, Fujisawa, Kanagawa 252-0882, Japan

## Abstract

Kampo medicine is the Japanese adaptation of traditional medicine. In Kampo medicine, “medical interview” plays an important role. “Medical interview” in Japanese traditional medicine includes not only chief complaint but also a questionnaire that asked about the patient's lifestyle and subjective symptoms. The diagnosis by Kampo is called “Sho” and determined by completely different view from Western medicine. Specialists gather all available information and decide “Sho.” And this is the reason why non-Kampo specialists without technical knowledge have difficulties to use traditional medicine. We analyzed “medical interview” data to establish an indicator for non-Kampo specialist without technical knowledge to perform suitable traditional medicine. We predicted “Sho” by using random forests algorithm which is powerful algorithm for classification. First, we use all the 2830 first-visit patients' data. The discriminant ratio of training data was perfect but that of test data is only 67.0%. Second, to achieve high prediction power for practical use, we did data cleaning, and discriminant ratio of test data was 72.4%. Third, we added body mass index (BMI) data to “medical interview” data and discriminant ratio of test data is 91.2%. Originally, deficiency and excess category means that patient is strongly built or poorly built. We notice that the most important variable for classification is BMI.

## 1. Introduction

Interest in traditional medicine has increased nowadays. People in many countries look to traditional medicine to maintain their health or cure their disease. The WHO Western Pacific Regional Office (WHO WPRO) published “WHO International Standard Terminologies on Traditional Medicine (TM) in the Western Pacific Region (WHO ISTT)” in 2007 and linked the modern and traditional medicines together. The World Health Organization (WHO) suggests the integration of traditional medicine into the next edition of the International Statistical Classification of Diseases and Related Health Problems (ICD-11) [1–4].

Kampo medicine—the Japanese adaptation of traditional medicine—was developed uniquely. Since 1967, the Japanese government has included Kampo medicine in the national medical system. In 2013, a total of 149 Kampo drugs are approved as prescription drugs and covered by the public insurance. Uniqueness of Kampo medicine is in that it has been a traditional medicine integrated into a public health care system in conjunction with modern medicine, while traditional medicines in other countries, for example, China and Korea, are often separated from modern medicine [5–9].

In Japanese traditional medicine, there are some types of examinations: “medical interview,” tongue diagnosis, audioolfactory assessment, abdominal diagnosis, palpation, and so on. Kampo specialists consider all the various factors together. In particular, “medical interview” plays an important role. “Medical interview” includes not only chief complaint but also a questionnaire that asked about the patient's lifestyle and subjective symptoms which are seemingly unrelated to chief complaint. Specialists will gather all available information and decide “Sho” and traditional herbal medicine. The diagnosis by Kampo is called “Sho” and determined by completely different view from Western medicine. The diagnosis by Kampo is described by combination of categories such as deficiency and excess, cold and heat, Qi, blood, and fluid. Each category has some patterns of “Sho.” In deficiency and excess category, there are five “Sho:” deficiency pattern, slightly deficiency pattern, between-deficiency-and-excess pattern, slightly excess pattern, and excess pattern. Also, in cold and heat category, there are three patterns of “Sho”: cold pattern, between cold and hot pattern, and hot pattern. And in Qi, Blood, and Fluid categories, there are 6 patterns of “Sho”: Qi-deficiency pattern, Qi-counterflow pattern, Qi-depression pattern, blood-deficiency pattern, Oketsu pattern, and fluid-disturbance pattern [10]. When the patient is cold in Western diagnosis, the diagnosis by Kampo is, for example, deficiency pattern, between cold and hot pattern, or Qi-counterflow. The same “Sho” does not always describe the same Western diagnosis. Even if the patient has the same Western diagnosis, “Sho” is different among patients. In order to determine herbal medicine or “Sho,” technical knowledge and experience are required. For non-Kampo specialist, it is hard to connect result of “medical interview” and “Sho”.

In this paper, we focus on deficiency and excess category. If the repairing responses shown by the patient against his/her disease condition are strong or fully active, the patient is said to be in excess pattern, while, if they are weak or hollow, they are said to be in deficiency pattern [10]. Moreover deficiency and excess category means that patient is strongly built or poorly built. We analyze “medical interview” data to establish an indicator for non-Kampo specialist without technical knowledge to perform suitable traditional medicine.

## 2. Subjects and Methods

Since 2006, Center of Kampo Medicine, Keio University School of Medicine, has collected data about patients' “medical interview,” “Sho,” Western disease name (ICD-10 code), and prescribed herbal medicine. From April 2006 till December 2011, we collected 16805 records which include return to clinic records, and the number of first-visit patients that we analyzed was 2830. All registered patients provided written informed consent. Patients enter “medical interview” information via touch panel operation. “Medical interview” has 362 items, ranges in content from physical sign to food preference, and is important for Kampo diagnosis. We use patients' 128 subjective symptoms. There are two types of questions, yes-no (24 items) questions and Visual Analogue Scale (VAS) questions (104 items). The Visual Analogue Scale (VAS) has been developed to allow the measurement of individual's responses to physical stimuli, such as heat. The VAS is a method that can be readily understood by most people to measure a characteristic or attitude that cannot be directly measured. It was originally used in the field of psychometrics, and nowadays it is widely used to assess changes in patient health status with treatment. A VAS consists of a line on a page with clearly defined end points and normally a clearly identified scale between the two end points. For guidance, the phrases “no pain” and “worst imaginable pain” are placed at both sides of the line, respectively. Minimum values 0 of the VAS means “no pain” and maximum values 100 means “worst imaginable pain.” In this paper, we use normalized VAS. To get normalized VAS, we divided VAS by each patients' maximum VAS value [11].

We predict “Sho” by using 2830 first-visit patients' “medical interview” data. In this paper, we focus on deficiency and excess category as a target and adopt random forests algorithm. In deficiency and excess category, there are five “Sho”: deficiency pattern (437 patients), slightly deficiency pattern (395 patients), Between-deficiency-and-excess pattern (1500 patients), slightly excess pattern (268 patients), and excess pattern (230 patients). These data were diagnosed by Doctors of Center of Kampo Medicine, Keio University School of Medicine, who are Kampo specialist.

Random forests algorithm was proposed by Breiman [12, 13] and is an algorithm for classification that uses an ensemble of classification trees. Random forests algorithm has performance in classification tasks, comparable to support vector machines. It is unexcelled in accuracy among current algorithms, can handle thousands of input variables without variable deletion, and gives estimates of which variables are important in the classification. The overview of random forests algorithm is as follows: random forests algorithm grows many classification trees. To classify a new object from an input vector, put the input vector down each of the trees in the forest. Each tree gives a classification, and we say that the tree “votes” for that class. The forest chooses the classification having the most votes. We set training and test data that has labels consistent with that type of classification. All statistical analyses were conducted using R software, version 2.15.2 (The R Foundation for Statistical Computing; October 26, 2012), on Mac OS X10.7.5 powered by 3.2 GHz Quad-Core Intel Xeon.

## 3. Results

### 3.1. 2830 First-Visit Patients' Profiling

The mean age was 46.7 ± 18.6 years. With regard to sex, there were 814 men and 2016 women.

### 3.2. All Data

**Figure 1:**
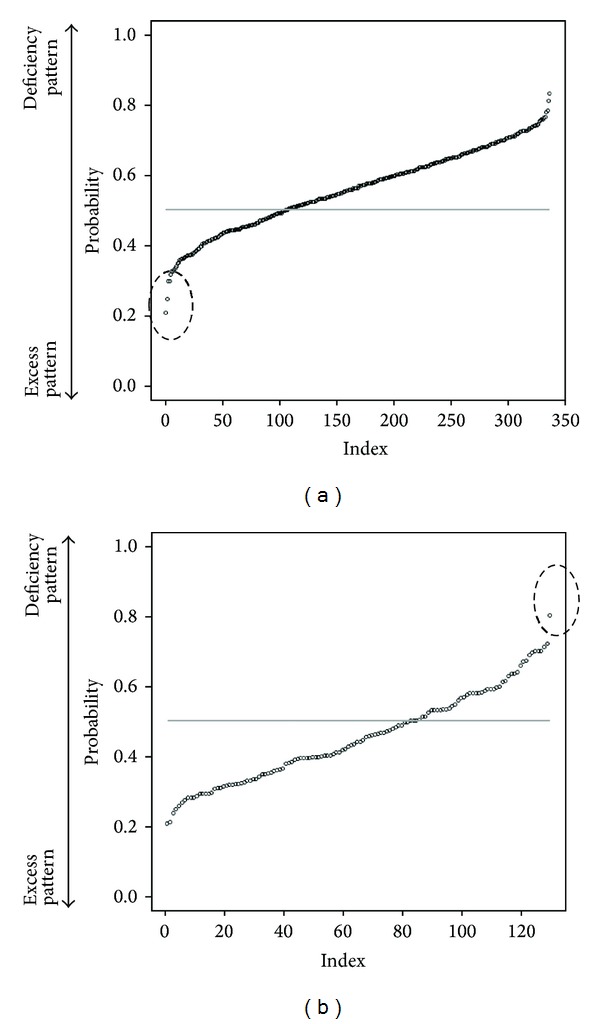
Each patient's prediction probability of test data. (a) label is deficiency pattern (b) label is exess pattern. (a) plotted probability of deficiency pattern test patients data (point means each patient) and the closer value of 1 means that the patient is of deficiency pattern and the closer value of 0 means excess pattern. So if each point is above horizon line = 0.5, the point is classified into deficiency pattern. (b) plotted probability of excess pattern test patients' data (point means each patient) and the closer value of 1 means that the patient is of deficiency pattern and the closer value of 0 means excess pattern. So if each point is below horizon line = 0.5, the point classified is into excess pattern. We can notice some outlier predictions in circles. They answered few “medical interviews”.

We selected randomly 200 patients as a training data (each 100 patients from deficiency pattern and excess pattern). And others are test data. The discriminant ratio of training data was perfect but that of test data is 67.0% (Table 1). In Figure 1, points are patients' prediction probabilities. The closer value of 1 means that the patient is deficiency pattern and the closer value of 0 means excess pattern. If above 0.5, we estimate that he is deficiency pattern. If below 0.5, he is excess pattern.

### 3.3. After Data Cleaning

**Figure 2:**
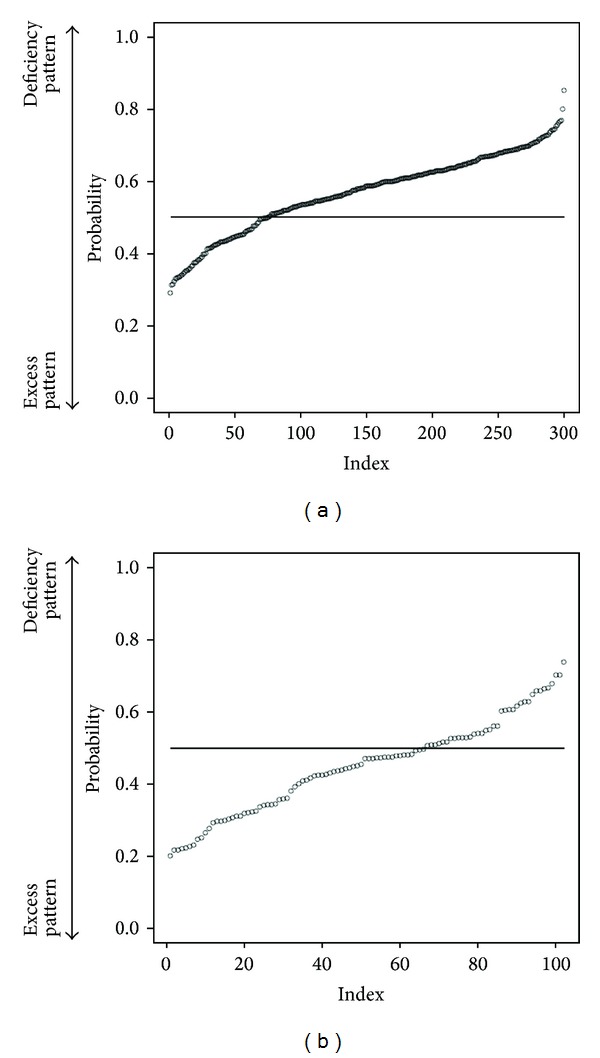
Each patient's prediction probability of test data answered more than 20 items of “medical interview”. (a) plotted probability of deficiency pattern patients' data (point means each patient) and the closer value of 1 means that the patient is of deficiency pattern and the closer value of 0 means excess pattern. So if each point is above horizon line = 0.5, the point is classified into deficiency pattern. (b) plotted probability of excess pattern patients' data (point means each patient) and the closer value of 1 means that the patient is of deficiency pattern and the closer value of 0 means excess pattern. So if each point is below horizon line = 0.5, the point is classified into excess pattern. We notice that there are no outlier predictions.

**Figure 3:**
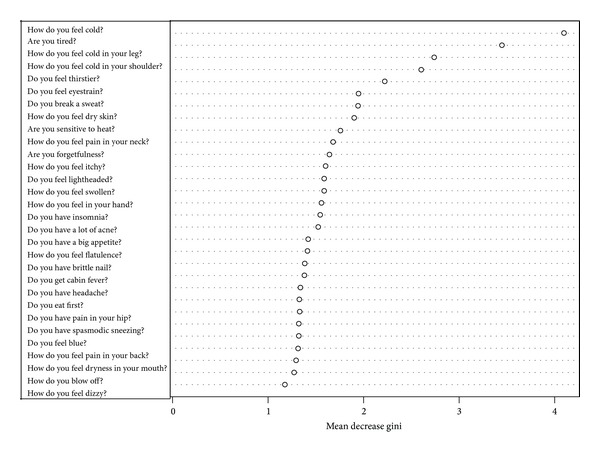
Top 30 important variables answered more than 20 items of “medical interview”. Higher value of mean decrease gini means that the item makes a sizable contribution to predict the “Sho.”

To get prediction power enough for practical use, we try to do data cleaning. From 2830 first-visit patients, we choose patients who answered more than 20 items of “medical interview.” The number of target first-visit patients is 2540: deficiency pattern (400 patients), slightly deficiency pattern (364 patients), Between-deficiency-and-excess pattern (1335 patients), slightly excess pattern (239 patients), and excess pattern (202 patients). We selected randomly 200 patients as a training data (each 100 patients from deficiency pattern and excess pattern). And others are test data. The discriminant ratio of training data was also perfect and that of test data is 72.4% (Table 2). In Figure 2, points are patients' prediction probabilities. We tried to use Slightly Deficiency pattern and Slightly Excess pattern data as test data. Discriminant ratio was 63.8% (Table 3). Important variables in this classification are in Figure 3.

### 3.4. Predict of “Sho” with Body Mass Index

**Figure 4:**
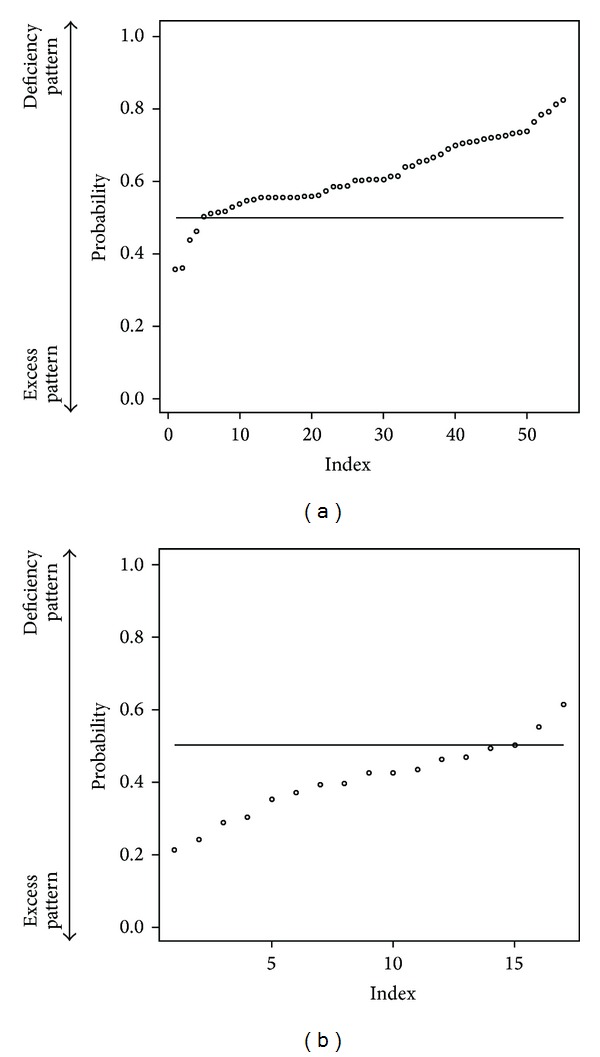
Each patient's prediction probability of test data. Predict with BMI. (a) label is deficiency pattern. (b) label is excess pattern. (a) plotted probability of deficiency pattern test patients' data (point means each patient) and the closer value of 1 means that the patient is of deficiency pattern and the closer value of 0 means excess pattern. So if each point is above horizon line = 0.5, the point is classified into deficiency pattern. (b) plotted probability of excess pattern test patients' data (point means each patient) and the closer value of 1 means the patient is of deficiency pattern and the closer value of 0 means excess pattern. So if each point is below horizon line = 0.5, the point is classified into excess pattern.

**Figure 5:**
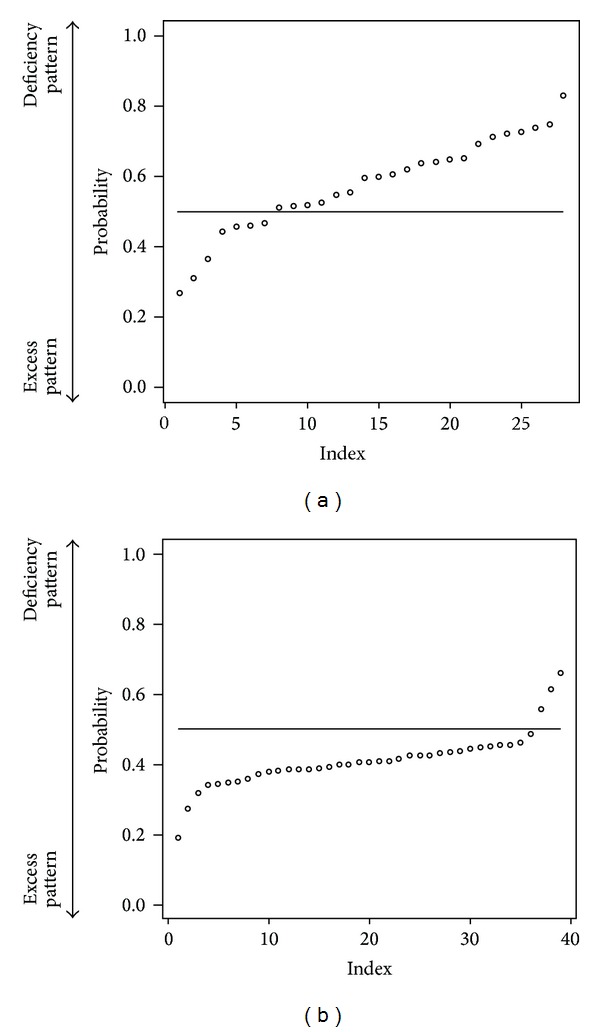
Each patient's prediction probability of test data. Predict with BMI. (a) label is slightly deficiency pattern. (b) label is slightly excess pattern. (a) plotted probability of slightly deficiency pattern test patients' data (point means each patient) and the closer value of 1 means that the patient is of deficiency pattern and the closer value of 0 means excess pattern. So if each point is above horizon line = 0.5, the point is classified into deficiency pattern. (b) plotted probability of slightly excess pattern test patients' data (point means each patient) and the closer value of 1 means that the patient is of deficiency pattern and the closer value of 0 means excess pattern. So if each point is below horizon line = 0.5, the point is classified into excess pattern. The patients' prediction probabilities are higher in Figure 5(a) and lower in Figure 5(b).

**Figure 6:**
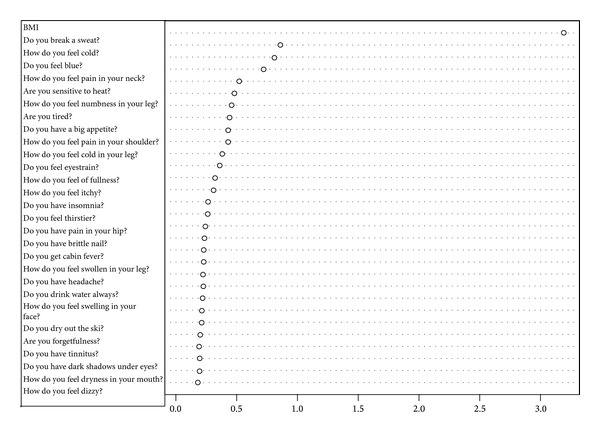
Top 30 important variables. Predict with BMI. Higher value of mean decrease gini means that the item makes a sizable contribution to predict the “Sho.” We notice that the most important variable is BMI.

We added body mass index (BMI) data to “medical interview” data. Center of Kampo Medicine, Keio University School of Medicine, has patients' BMI data of 2011. The number of target first-visit patients is 402: Deficiency pattern (75 patients), Slightly deficiency pattern (28 patients), Between-deficiency-and-excess (223 patients), Slightly excess pattern (39 patients), and Excess pattern (37 patients). We selected randomly 40 patients as a training data (each 20 patients from deficiency pattern and excess pattern). And others are test data. The discriminant ratio of training data was perfect and that of test data is 91.2% (Table 4). In Figure 4, points are patients' prediction probabilities. We try to use slightly deficiency pattern and slightly excess pattern data as test data. Discriminant ratio was 85.1% (Table 5). In Figure 5, points are patients' prediction probabilities. Important variables in this classification are in Figure 6. The most important variable is BMI.

## 4. Discussion

**Figure 7:**
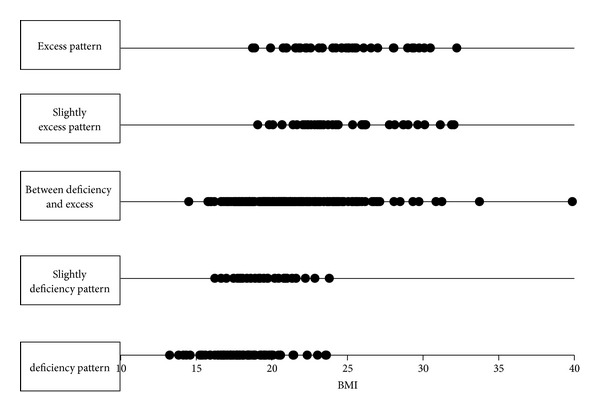
Relationship between BMI and deficiency and excess category. This is not a linear relationship, so it is hard to describe the relationship with BMI and deficiency and excess category by linear approximation.

We predicted “Sho” by using random forests algorithm which is a powerful algorithm for classification. First, we used all the 2830 first-visit patients' data. The discriminant ratio of training data was perfect but that of test data is only 67.0%. In Figures 1(a) and 1(b), we can notice some outliers in red circles. It was problem of data itself. Some people answer few “medical interview questions”. This data is meaningless, and prediction failed. So we did data cleaning to get prediction enough for practical use. We choose patients who answered more than 20 items. In this situation, the discriminant ratio of training data was also perfect and that of test data is 72.4% (Table 2). It was slightly better than first situation. And we notice that there are no outlier predictions in Figure 2. However, it was not an acceptable result. Originally, deficiency and excess category means that patient is strongly built or poor built and our “medical interview” did not include such indicator. In Figure 3, there are no items by which we can determine someone's appearance. Our “medical interview” questionnaire did not take patient's appearance into account, so this prediction with random forests did not work well. If we had chosen another statistical strategy, we would obtain almost the same results, because our questionnaire lacks appropriate questions that ask about body appearance. The relationships among deficiency, excess categories, and BMI are in Figure 7; however, it is not, linear relationship. If we use only BMI for classification, discriminant ratio was just 62.0%.

To cover the shortcomings of our questionnaire, we added BMI data to “medical interview” data. BMI is a simple index of weight-for-height that is commonly used to classify underweight, overweight, and obesity in adults. It is defined as the weight in kilograms divided by the square of the height in meters. The discriminant ratio of test data was 91.2%. We notice that patients' prediction probabilities are higher in Figure 4(a) and lower in Figure 4(b). It provided better discriminant ratio than the others. From Figure 3, we notice that the most important variable for classification is BMI. To get better classification, we have to know what is the feature of the target and check the data. In our result, BMI contributed to predict the “Sho” highest level than the other; however, by only using BMI prediction is flimsy. When non-Kampo specialists try to diagnose Deficiency and Excess category, they should first pay attention to patients' BMI. After that, non-Kampo specialists should ask some questions in medical examination. The questions also have higher contribution to predicting the “Sho”: Do you break a sweat? How do you feel cold? Do you feel blue? How do you feel pain in your neck? Are you sensitive to heat? How do you feel numbness in your leg? Are you tired? Do you have a big appetite? How do you feel pain in your shoulder? These questions indicate that a patient seems to be of deficiency pattern if the answer is yes. Of course there are many kinds of “medical interviews”; we think that the smaller number of questions is good for non-Kampo specialists, and it is enough for practical use.

## 5. Conclusion

In Japanese traditional medicine, Kampo, “medical interview” plays an important role. “Medical interview” is a questionnaire that asked about the patient's lifestyle and subjective symptoms. The diagnosis by Kampo is called “Sho” and determined by completely different view from Western medicine. And this is the reason why non-Kampo specialists without technical knowledge have difficulties to use traditional medicine. In addition, the diagnosis by Kampo that is called “Sho” is problem of increasing complexity because “Sho” is used in combination. We predicted “Sho” by using random forests algorithm which is powerful algorithm for classification. In our result, BMI is of higher contribution; however, if we only use BMI to predict “Sho” in deficiency and excess category, the discriminant ratio was 62.0%. To get higher-accuracy prediction, we should use both BMI and “medical interviewv” It is applied to non-Kampo specialists as well, so they try to use BMI and some “medical interview” which has higher contribution.

In this research, prediction of Deficiency and Excess category is enough for practical use if we added body mass index (BMI) data to “medical interview” data. Other categories are remained and are our future targets.

## Figures and Tables

**Table 1:** All data results of test data.

	Deficiency pattern	Excess pattern	Discriminant ratio
Predict			
Deficiency pattern	231	48	67.00%
Excess pattern	106	82	

Total	337	130	

**Table 2:** After data cleaning result of test data.

	Deficiency pattern	Excess pattern	Discriminant ratio
Predict			
Deficiency pattern	225	36	72.40%
Excess pattern	75	66	

Total	300	102	

**Table 3:** After data cleaning result of slightly deficiency pattern and slightly excess pattern of test data.

	Slightly Deficiency pattern	Slightly Excess pattern	Discriminant ratio
Predict			
Deficiency pattern	244	92	63.80%
Excess pattern	120	147	

Total	364	239	

**Table 4:** Predict with BMI result of test data.

	Deficiency pattern	Excess pattern	Discriminant ratio
Predict			
Deficiency pattern	51	2	91.20%
Excess pattern	4	15	

Total	55	17	

**Table 5:** Predict with BMI result of slightly deficiency pattern and slightly excess pattern of test data.

	Slightly deficiency pattern	Slightly excess pattern	Discriminant ratio
Predict			
Deficiencypattern	21	3	85.10%
Excess pattern	7	36	

Total	28	39	
